# Peripheral arterial disease and its correlates in patients with type 2 diabetes mellitus in a teaching hospital in northern Nigeria: a cross-sectional study

**DOI:** 10.1186/s12872-020-01395-3

**Published:** 2020-02-28

**Authors:** Orighomisan Freda Agboghoroma, Fatai Momodu Akemokwe, Fabian H. Puepet

**Affiliations:** 1 Jos University Teaching Hospital, Jos, Plateau State Nigeria; 2 Present address: Medical Research Council at the London School of Hygiene and Tropical Medicine, Fajara, Gambia

**Keywords:** Peripheral arterial disease, Diabetes mellitus, Type 2, Correlates, Nigeria

## Abstract

**Background:**

Peripheral arterial disease (PAD) is a risk factor for diabetic foot ulcer, limb amputation as well as coronary heart disease. It is more common in patients with diabetes mellitus (DM) and co-exists with peripheral neuropathy. Prevalence of PAD in type 2 DM patients in northern Nigeria is largely unknown. We investigated the occurrence and factors associated with PAD among patients with type 2 DM in a tertiary hospital in northern Nigeria.

**Methods:**

This was a cross- sectional analytic study. We recruited 200 patients with type 2 DM consecutively from the diabetes clinic of the Jos University Teaching Hospital. Ankle brachial index was assessed for each participant. Clinical information, anthropometric indices and blood samples were collected for assay. Data was analysed using CDC Epi-Info and logistic regression analysis was used to determine independent correlates of PAD.

RESULTS:PAD was present in 38.5%(*n* = 77) of subjects and it was associated with the female sex, age ≥ 50 years, Body mass index (BMI) ≥ 25 kg/m^2^ and low HDL cholesterol levels. However, on multiple logistic regression, a BMI ≥ 25 kg/m^2^ and a low HDL cholesterol level were independent correlates of PAD(adjusted OR = 2.13,95% CI = 1.04–4.36 and adjusted OR = 2.31, 95% CI = 1.04–5.15, respectively).

**Conclusion:**

PAD is present in more than a third of patients with type 2 DM in a tertiary hospital in northern Nigeria. A BMI of ≥25 kg/m^2^ and low HDL cholesterol levels were independent correlates of PAD.

## Background

Peripheral arterial disease (PAD) is a chronic complication of diabetes mellitus (DM) and a risk factor for foot ulceration and amputation- more than two-thirds of patients with diabetic foot ulceration have associated PAD [1]. This arthero – occlusive disease of the extremities does increase the risk of coronary artery disease as well [2, 3]. Although PAD occurs in non- diabetic patients, it is up to four times more common in patients with DM [4]. Symptoms such as intermittent claudication and calf pain at rest suggest PAD but in DM, these symptoms may be obscured by the concomitant presence of peripheral neuropathy [5].

The prevalence of PAD differs depending on the modality used [6]. Studies have used the presence of symptoms, the absence of peripheral pulses in the extremities and more recently, ankle brachial index (ABI) to assess PAD. In patients with DM, the presence of PAD as determined by ABI varies from 9 to 20% [3, 7, 8] in Western countries and from 3.2–24.1% [9–12] in Asia. In sub- Saharan Africa, recent studies on PAD in patients with DM estimate the occurrence of PAD to be between 28 and 52.5% using ABI [13–15]. Ankle brachial pressure index, a ratio of the systolic blood pressure in the ankle to the systolic pressure in the arm, has 95% specificity and 100% sensitivity for PAD at a value of ≤0.90 [16, 17]. The American Diabetes Association recommends screening for PAD in DM patients with ABI and treatment to prevent further morbidity [2].

In general, the risk factors for PAD include DM, hypertension, smoking, age, obesity and dyslipidaemia [18]. However, in patients with DM there are other factors associated with PAD: hyperglycaemia and a longer duration of diabetes [2, 19]. The major difference in the pathogenesis of atherosclerosis in DM lies in the effects of chronic hyperglycaemia. Prolonged hyperglycaemia results in augmentation of platelet activity and alterations in the vascular endothelium and coagulating factors, which favours atherosclerosis and thrombosis [19–21]. Hence, searching for risk factors related to PAD and modifying them where possible may prove helpful in the prevention and management of this condition.

Few studies from sub-Saharan Africa fully examine PAD [13, 14, 22]. An in-depth knowledge of PAD in African patients with DM is necessary. Therefore, our study focuses on determining the prevalence of PAD in patients with type 2 DM with emphasis on the clinical and biochemical factors associated with it.

## Methods

This is a cross- sectional analytic study carried out at the Diabetes Clinic of the Jos University Teaching Hospital. The hospital is located in the north central Nigeria and is the main tertiary institution in the region providing referral services to seven neighbouring states with a bed capacity of 520. We obtained ethical approval for the study from the institution’s Health Research Ethical Committee (JUTH/DCS/ADM/127/XIX/5955). Participants were recruited consecutively, from March to September in 2014, after providing an informed consent. We included those who were: [1] diagnosed with DM and on anti-diabetic therapy, [2] 30 years and older at the time of diagnosis (type 2 DM was more likely in this age group) and, [3] not on steroids or oral contraceptives. The minimum sample size was calculated using a PAD prevalence of 52.5% [23] among patients with DM and a precision of 0.1. However, 200 patients were enrolled to increase the statistical power of the study tests. A proforma was used to collect all the data required for the study including biodata, medications, anthropometric measurements and biochemical characteristics.

The weight and height for each patient were measured using a weighing scale and a stadiometer (Seca© 224, United Kingdom). Weight was recorded in kilogrammes (to the nearest 0.1 kg) and height in metres (to the nearest 0.01 m). The Body Mass Index (BMI) was calculated as the weight of a participant divided by the square of his/her height. Waist circumference was measured using a non- stretch measuring tape and at a position midway between the costal margin and the iliac crest. An abnormal waist circumference was defined as ≥94 cm for men and ≥ 80 cm for women [24]. Glycosylated haemoglobin(HbA1c) was measured using the a latex agglutination method in the DCA 2000 analyser (Siemens, Germany) and the lipid levels were estimated using a blood chemistry autoanalyser (Hitachi 902, Mannheim, Germany).

Ankle and brachial pressures were measured using a mercury manometer (Accosons, England) and a 8-MHz handheld vascular doppler device (LifeDop L150R, Colorado,United States). The highest systolic pressures for each limb were used to calculate the ABI with the ankle systolic pressure as the numerator and the brachial systolic pressure as the denominator. An ABI of ≤0.90 in any limb was recorded as PAD. PAD was further classified into mild, moderate and severe using the ABI, as follows: mild − 0.70 – 0.90; moderate – 0.40 – 0.69 and severe- less than 0.40 [17, 25].

Hypertension was present if a participant was on antihypertensives or had a blood pressure of ≥140/90 mmHg [26]. Poor glycaemic control was defined as HbA1c ≥ 7% [27]. Dyslipidaemia was described as follows: elevated total cholesterol – total cholesterol value ≥5.2 mmol/L or Elevated Triglyceride- triglyceride level ≥ 1.7 mmol/L or Elevated low density lipoprotein(LDL) Cholesterol -LDL cholesterol ≥2.6 mmol/L [28]. Low High Density cholesterol was defined as values < 1.1 mmol/L for men and < 1.3 mmol/L for women [28].

Data was entered and analysed using the CDC Epi-info software package. Comparisons between continuous variables was done using t- test and categorical variables using chi-square. Bivariate analysis was used to determine the relationship between PAD and other variables. Multiple logistic regression was used to determine the independent correlates of PAD- the independent variables were those which showed statistically significant associations with PAD on bivariate analysis in this study. A *p*-value of less than 0.05 was considered statistically significant.

## Results

The age of participants ranged between 31 and 90 years. The prevalence of PAD was 38.5% (*n* = 77). Mild and moderate PAD was seen in 49(24.5%) and 23(11.5%), respectively while one participant had severe PAD. Twenty (10%) patients had poorly compressible arteries with ankle – brachial index of > 1.3.

There were 83 (41.5%) female participants in the study. Most of the participants were middle aged and about two- thirds had systemic hypertension in addition to DM (Table 1). Nine (4.5%) participants had previously smoked tobacco but none was currently smoking. The anthropometric measurements are shown in Table 1: the women had a higher BMI than the men and this reached statistical significance. The males and females also differed in their cholesterol levels: the females had significantly higher total cholesterol and LDL cholesterol levels.

**Table 1:** Clinical and biochemical characteristics of study participants in mean (SD) or n (%)

Characteristic	Female (*n* = 83)	Male (*n* = 117)	*p* value	All participants(*n* = 200)
Age (years)	56.5 (12.5)	57.4 (11.3)	0.65	56.9 (11.8)
Duration of DM (years)	9.2 (6.9)	8.3 (6.8)	0.39	8.7 (6.8)
Presence of Hypertension	61 (73.5)	79 (67.5)	0.36	140 (70.0)
Previous Tobacco Smoking*	0 (0.0)	9 (7.7)	0.01	9 (4.5)
Previous MI or stroke	4 (4.8)	8 (6.8)	0.55	12 (6.0)
Medications				
Antihypertensives	60 (72.3)	74 (63.2)	0.18	134 (67.0)
Lipid lowering drugs	11 (13.3)	14 (12.0)	0.79	21 (12.5)
Antiplatelets	15 (18.1)	22 (18.8)	0.90	37 (18.5)
BMI (Kg/m^2^)^*^	29.3 (5.4)	27.0 (5.1)	< 0.01	27.9 (5.4)
Waist circumference (cm)	97.6 (9.9)	94.6 (12.2)	0.05	95.8 (11.4)
HbA1c (%)	10.3 (3.1)	9.9 (3.1)	0.32	10.1 (3.1)
Total cholesterol (mmol/L)^*^	4.5 (0.9)	4.1 (1.0)	0.01	4.20 (1.0)
LDL cholesterol (mmol/L)	2.7 (0.9)	2.5 (0.9)	0.14	2.6 (0.5)
HDLcholesterol (mmol/L)*	1.0 (0.4)	0.9 (0.4)	< 0.01	0.95 (0.4)
Triglycerides (mmol/L)	1.5 (0.7)	1.6 (1.1)	0.65	1.5 (0.9)
PAD*	39 (47.0)	38 (32.5)	0.04	77 (38.5)

*p < 0.05. *DM* Diabetes mellitus, *MI* Myocardial infarction, HbA1c glycosylated haemoglobin, *BMI* Body mass index, *LDL* Low density lipoprotein, *HDL* High density lipoprotein, *PAD* Peripheral arterial disease

On bivariate analysis for the correlates of PAD, more females had PAD than males and this was statistically significant (Table 2). Persons who were 50 years and older were less likely to have PAD (*p* < 0.05). Among those with PAD, a large proportion had hypertension, abnormal waist circumference and poor glycaemic control – 49 (63.6%), 53(68.8%) and 61(79.2%), respectively but these did not attain statistical significance. A BMI of ≥25 kg/m2 and a low HDL cholesterol was associated with the presence of PAD in this study on bivariate analysis. There was an independent association between BMI of 25 kg /m^2^ or more, low HDL cholesterol levels and PAD on multiple logistic regression with age and sex as co-variates (Table 3). The regression model predicted 11% of the variance in PAD and correctly classified 68% of cases.

**Table 2:** Comparison of clinical and biochemical characteristics of participants with PAD

Characteristic	PAD Absent n (%)	PAD present n (%)	Odds ratio	95% Confidence interval
Age ≥ 50 years^*^	97 (78.9)	50 (64.9)	0.50	0.26–0.94
Female sex^*^	44 (35.8)	39 (50.6)	1.84	1.04–3.29
Duration of DM > 5 years	74 (60.2)	38 (49.4)	1.55	0.87–2.75
Presence of hypertension	91 (74.0)	49 (63.6)	0.62	0.33–1.14
Previous MI or Stroke	7 (5.7)	5 (6.5)	1.15	0.35–3.76
Previous tobacco smoking	5 (4.1)	4 (5.2)	0.77	0.20–2.97
Medications				
Antihypertensives	87 (70.7)	47 (60.1)	0.65	0.36–1.18
Lipid lowering drugs	16 (13.0)	9 (11.7)	0.89	0.37–2.12
Antiplatelets	28 (22.8)	9 (11.7)	0.47	0.20–1.06
BMI ≥25 kg/m^2*^	77 (62.6)	63 (81.8)	2.69	1.35–5.33
Abnormal Waist Circumference	82 (66.7)	53 (68.8)	1.10	0.60–2.03
Poor glycaemic control	101 (82.1)	61 (79.2)	0.83	0.40–1.70
Elevated total cholesterol	20 (16.3)	13 (16.9)	1.05	0.49–2.25
Elevated LDL cholesterol	56 (45.5)	43 (55.8)	1.50	0.85–2.60
Low HDL cholesterol^*^	89 (72.4)	64 (85.3)	2.22	1.05–4.72
Elevated Triglycerides	32 (26.0)	21 (27.3)	1.07	0.56–2.03

^*^p < 0.05. *DM* Diabetes Mellitus, *MI* Myocardial infarction, *PAD* Peripheral arterial disease, *BMI* Body mass index, *LDL* Low density lipoprotein, *HDL* High density lipoprotein

**Table 3:** Adjusted odds ratios for independent correlates of PAD using multiple logistic regression analysis

Characteristics	Adjusted odds ratio	95% Confidence Interval
Female sex	1.57	0.85–2.91
Age ≥ 50 years	0.60	0.31–1.17
BMI ≥ 25 kg/m^2*^	2.13	1.04–4.36
Low HDL cholesterol*	2.31	1.04–5.15

^*^p < 0.05. *BMI* Body mass index, *HDL* High density lipoprotein

## Discussion

Peripheral arterial disease was common in patients with type 2 DM in this study: 38.5% of our DM patients had PAD. Our findings are comparable to studies in Southern Nigeria where similar populations were assessed for PAD [13, 15]. One reason for the high prevalence of PAD in our study is that DM co- exists with other risk factors for cardiovascular disease. We observed that more than two-thirds of the patients in this study had hypertension, dyslipidaemia, and were overweight or obese. Secondly, it is likely that patients with DM who present at the teaching hospital already have chronic complications of DM hence their referral. Earlier studies from sub Saharan Africa had shown a lower prevalence mainly on account of the use of symptoms and signs as proxies for estimating PAD [6]. Our results differ from studies done in Asia, US and Europe, in that the recorded prevalence in those studies were considerably lower- as low as 3.2% in a similar population in Korea [9]. A high proportion of other risk factors for PAD, such as hypertension, in Black Africans may explain the differences in the prevalence PAD in this case. Perhaps the combination of all the risk factors present in our patients resulted in our observation. Ten percent of our patients had poorly compressible arteries (ABI > 1.3). Adding this number to the prevalence of PAD increases the prevalence to 48.5%.

More females than males had PAD in this study. This was likely due to significantly increased BMI and total cholesterol levels in the female participants. Rhee et al. (2007) and Okello et al. (2014) observed a female preponderance that was statistically significant [11, 22]. Other studies had similar findings that were not statistically significant [12, 14, 23]. In one study, PAD was more common in males because there was a higher proportion of smokers [9]. Smoking was not common among our subjects- there were no current smokers and previous smoking was insignificant. Other studies we reviewed, found no relationship between sex and the PAD [10, 29].

We observed that older DM patients were less likely to have PAD and there was no relationship between the duration since diagnosis of DM with PAD. Perhaps other risk factors were more common in the younger diabetics such as dyslipidaemia. Mwebaze et al. (2014) and Ogbera et al. (2015) found that PAD was more common in younger DM patients, although this was not significant [14, 15]. This was different from the observations made in other studies [9, 29]. Advancing age as well as a longer duration of DM was associated with PAD in some studies in sub-Saharan Africa and elsewhere [10, 13, 23]. It is our theory that in the face of multiple risk factors for PAD including persistent hyperglycaemia and dyslipidaemia, the role of age or a longer duration of DM may be less substantial.

Hyperglycaemia plays a significant role in the pathogenesis of PAD in terms of increasing inflammation, disrupting platelet function and the vascular endothelium as well as altering blood rheology [19]. However, in our study we found no significant relationship between poor glycaemic control as represented by HbA1c ≥ 7% and PAD. Rather, the mean HbA1c was greater in the absence of PAD. This was similar to what Ogbera et al. (2015) observed their study [15]. In contrast, there was a significant association between hyperglycaemia and PAD in studies from Asia [10, 11]. HbA1c is a snapshot of a patient’s glycaemic status over a three-month period: it may not be a true reflection of the glycaemic control in that individual over a longer period. At least one other study corroborates this [30].

Dyslipidaemia, in the form of low HDL cholesterol, was an independent correlate of PAD in this study. Few studies corroborate our findings [31]. In two of those studies, elevated total cholesterol and triglycerides were associated with PAD [9, 10]. Low HDL levels have been associated with atherosclerosis in general. Importantly, in contrast to the proportion of patients with dyslipidaemia, few patients were on lipid altering drugs.

Obesity as a risk factor for vascular disease played out in this study: most of the participants with PAD were overweight or obese, according to BMI. This was independent of other factors including dyslipidaemia and poor glycaemic control. This was similar to the findings in studies from Africa and the Middle East [29, 32]. A multi-country study in Asia found a relationship between lower BMI and PAD: few participants in the study were obese or overweight [11]. Truncal obesity, as defined by waist circumference did not correlate with PAD in our research. A likely explanation is that visceral adipose tissue in black Africans may not play a major role in the inflammatory changes seen in PAD [33, 34].

The findings in our study are limited to tertiary hospital settings- it is not applicable to the patients with diabetes in the general population. It is possible that there are other factors associated with PAD in patients with diabetes such as lifestyle choices and economic status, which we did not consider in our research. Exploration of the impact of these factors may prove important in future research on PAD in this population.

## Conclusion

More than a third of our patients with type 2 DM had PAD and this was associated with a younger age group(< 50 years), female sex, BMI and low HDL cholesterol levels. A BMI of ≥25kgm^2^ and low HDL cholesterol were independent correlates of PAD. Further investigation of the patients with ABI of > 1.3 may increase the prevalence. Screening for PAD in patients with DM will prove valuable in our practice. Maintaining a normal BMI and low HDL cholesterol for patients with DM may be key to reducing the occurrence of PAD.

## Data Availability

The datasets generated and analysed during the current study are not publicly available because they are still in use but are available from the corresponding author on reasonable request.

## Abbreviations

ABI: Ankle brachial pressure index
BMI: Body mass index
DM: Diabetes mellitus
HbA1c: Glycosylated haemoglobin
HDL: High density lipoprotein
LDL: Low density lipoprotein
OR: Odds ratio
PAD: Peripheral arterial disease
